# A Bitter Sweet Symphony: Immune Responses to Altered *O*-glycan Epitopes in Cancer

**DOI:** 10.3390/biom6020026

**Published:** 2016-05-03

**Authors:** Lenneke A.M. Cornelissen, Sandra J. Van Vliet

**Affiliations:** Department of Molecular Cell Biology and Immunology, VU University Medical Center, Amsterdam, 1081 HZ, The Netherlands; l.cornelissen@vumc.nl

**Keywords:** cancer, *O*-glycosylation, immunity, Siglec, C-type lectin

## Abstract

The appearance of aberrant glycans on the tumor cell surface is one of the emerging hallmarks of cancer. Glycosylation is an important post-translation modification of proteins and lipids and is strongly affected by oncogenesis. Tumor-associated glycans have been extensively characterized regarding their composition and tumor-type specific expression patterns. Nevertheless whether and how tumor-associated glycans contribute to the observed immunomodulatory actions by tumors has not been extensively studied. Here, we provide a detailed overview of the current knowledge on how tumor-associated *O*-glycans affect the anti-tumor immune response, thereby focusing on truncated *O*-glycans present on epithelial tumors and mucins. These tumor-associated *O*-glycans and mucins bind a variety of lectin receptors on immune cells to facilitate the subsequently induction of tolerogenic immune responses. We, therefore, postulate that tumor-associated glycans not only support tumor growth, but also actively contribute to immune evasion.

## 1. Introduction

Glycosylation is one of the most important post-translational modifications of proteins and lipids and it is estimated that over 95% of all cell surface proteins are elongated with glycans. Glycosylation plays a fundamental role in various cellular processes, including cell-cell recognition, cell-matrix interactions, as well as intracellular signaling. Glycans regulate the folding of newly-synthesized proteins and is, therefore, crucial for protein stability [1]. The glycosylation status of a cell is highly dynamic and dramatically alters upon oncogenesis, whereby cancer cells, compared to their healthy counterparts, express more branched *N*-glycans, higher levels of fucosylated and sialylated glycans and a truncated *O*-glycan phenotype [2]. This abundant and aberrant cancer glycosylation profile is currently widely accepted as a distinct hallmark of cancer. Although the immune system is capable of attacking and eradicating cancer cells, it is often suppressed within the tumor microenvironment. In this review we focus on the short *O*-glycans and highlight the current state of knowledge on the effects of these tumor-associated glycans on ongoing anti-tumor immune responses. We discuss how these truncated *O*-glycan structures, through their interaction with glycan-binding receptors on immune cells mislead anti-tumor immunity and, thereby, facilitate immune escape by the tumor.

## 2. Tumor-Associated *O*-glycosylation

**Figure 1:**
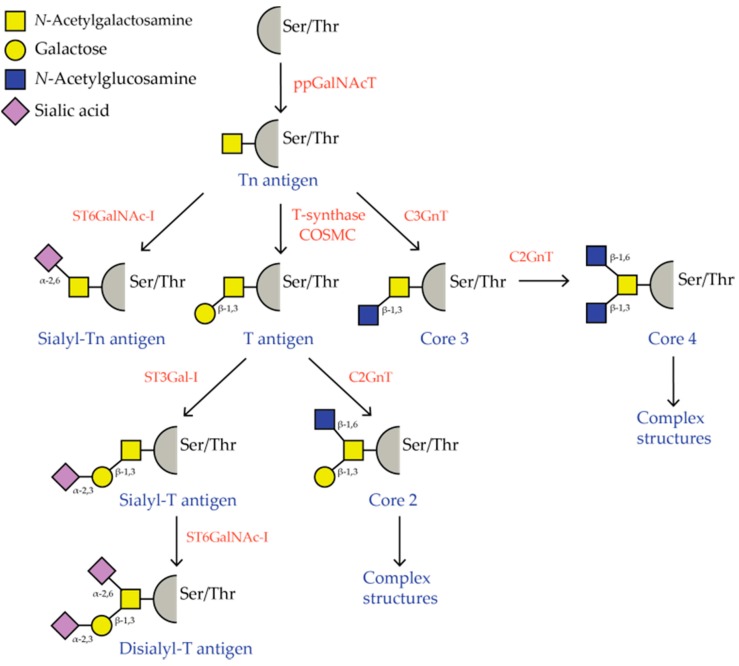
Overview of the *O*-GalNAc glycosylation pathway. Glycosyltransferases are shown in red and *O*-glycan structures in blue.

The *O*-glycosylation pathway starts with the addition of a single *N*-acetylgalactosamine (GalNAc) to a serine or threonine residue, thus forming the Tn antigen epitope. The Tn antigen can be further elongated with galactose to form the T antigen (also known as Core 1 or Thomsen-Friedenreich antigen) or with *N*-acetylglucosamine (GlcNAc), to form Core 3. *O*-glycans in healthy cells are generally even further extended to complex branched Core 2 or Core 4 structures, whereby the truncated Tn antigen is only expressed at high levels during embryogenesis. Complex Core 3 and Core 4 structures are exclusively present in the intestinal tract. In contrast to healthy cells, tumor cells are characterized by the appearance of truncated *O*-glycans, such as the Tn antigen and T antigen, on the cell surface. These truncated structures can be sialylated creating sialyl-Tn (sTn) and sialyl- of disialyl-T (sT), which prevents further elongation of the glycan structure. Two distinct sialyltransferases are responsible for the sialylation of truncated *O*-glycans, namely alpha-2,6-sialyltransferase ST6GalNAc-I [3,4] and alpha-2,3-sialyltransferase ST3Gal-I (Figure 1).

Synthesis of Tn antigen is mediated by an UDP-GalNAc:polypeptide GalNAc-transferase (ppGalNAcT) that transfers the α-GalNAc to a serine or threonine residue. Expression of ppGalNAcTs is dynamic and these enzymes have been shown to relocate from the Golgi to the ER in certain tumor types [5]. This relocation facilitates a prolonged mode of action, leading to enhanced expression of the Tn antigen on the tumor. The T antigen is formed through the action of the glycosyltransferase T-synthase (Core 1 β3-galactosyltransferase) and its chaperone COSMC [6,7]. The T-synthase and COSMC are expressed independently, however, COSMC is essential for T synthase function. Therefore, overexpression of COSMC may lead to an increase of T antigen, while a downregulation of COSMC may result in overexpression of Tn antigen. Thus, COSMC can affect the expression of truncated *O*-glycans and tumor progression on multiple levels. For breast cancer, COSMC function seems supportive for cancer progression, since loss of the T antigen-derived *O*-glycans decreases breast tumor development in mice [8]. In hepatocellular cancer, COSMC mRNA and protein are frequently overexpressed and this correlates with metastasis and poor survival [9]. Contradictory, knockdown of COSMC in the pancreatic cell line T3M4 actually induced oncogenic features with enhanced growth and tumor invasion [10]. Together, these results indicate that the impact of COSMC function and, thus, Tn or T antigen expression on oncogenesis, might be tumor type-specific, whereby, depending on the tumor type, either the Tn or T antigen plays a more dominant role.

The Tn and T antigens and their sialylated counterparts, sTn and sT antigens, are expressed by multiple tumor types, especially those of epithelial origin. Among these, expression levels of Tn antigen and T antigen seem quite comparable, ranging from 87.5% for breast cancer to 71% in ovarian cancer for Tn antigen and 87.5% for breast cancer, to 66% for ovarian cancer and 60% for colon cancer for T antigen [11,12,13]. The protein that carries T antigen depends on the tumor type, and includes several mucins and mucin-type proteins, such as CD44 on colorectal cancer, MUC1 on breast cancer, and CD164 in gastric and prostate cancer [14]. In gastric cancer, sTn is frequently expressed and modulates key mechanisms of cancer progression. As a consequence, sTn has become an indicator for poor prognosis [15], whereby ST6GalNAc-I is the major enzyme controlling sTn expression in gastric cancer [16]. Also bladder cancer is characterized by the overexpression of sTn, whereby 75% and 20% of the high- and low-grade tumors, respectively, express sTn. Correlation studies clearly point out that truncated *O*-glycans affect tumor progression, demonstrating that sTn expression is associated with a high risk of recurrence and progression in the high-grade bladder tumors [17]. Moreover, in lung adenocarcinomas Tn antigen is a significant and independent prognostic factor for overall and relapse-free survival [18]. The fact that tumor progression is based on clonal selection of the fittest cells within the heterogeneous cancerous population and that truncated *O*-glycans are specifically associated with malignant transformation, suggests that tumor cells expressing truncated *O*-glycans are preferentially selected, which likely promotes tumor cell survival. Indeed, colorectal carcinoma patients carrying T antigen positive tumors show a significantly higher risk to develop metastasis, as exemplified by increased expression of T antigen in liver metastasis (91%) compared to the primary tumor (60%) [11]. Although Tn and T antigen seem to act via different, tumor-type specific mechanisms, truncated *O*-glycans, in general, support tumor progression and their presence is strongly correlated to bad prognosis. For more insight into truncated *O*-glycans and tumor development, we refer the reader to an excellent and extensive review by Chia *et al.*[19].

Overall, the regulation of tumor-associated *O*-glycans, such as Tn, T antigen, and their sialylated counterparts, is complex and cannot be fully attributed to under- and overexpression of certain glycosyltransferases [20,21]. The strong correlation between tumor-associated *O*-glycans and bad prognosis points to the crucial role glycans and their mucin-type carrier proteins play in tumor progression and the development of metastasis. In addition, truncated *O*-glycans may contribute to tumor progression through evasion of anti-tumor immune responses. The broad impact of glycosylation on (cancer) cell function indicates that glycans not only play a fundamental role in cancer, but might also contribute to other more immune-related diseases (Box 1).

Box 1 **Aberrant O-glycosylation in other (immune-related) diseases.**

Aberrant O-glycans are involved in a variety of diseases, including autoimmunity and cancer, and demonstrate the multi-disciplinary action of glycans and the importance of glycosylation in maintaining health.

1. **IgA nephropathy (IgAN):** IgAN is a very common glomerulonephritis that is characterized by deposition of IgA immune complexes in the glomerulus. The O-glycans in the hinge-region of IgA-1 antibodies isolated from glomeruli deposits lack galactose and, thus, display high amounts of Tn antigen [22,23,24]. The aberrantly glycosylated IgA antibodies form immune complexes with anti-glycan autoantibodies, thus classifying IgAN as an autoimmune disease. The high quantity of Tn antigen on the IgA may facilitate binding to and subsequent signaling of the C-type lectin macrophage galactose-type lectin (MGL) on antigen presenting cells (APCs). MGL triggering has been shown to augment production of IL-10 [25], which could result in stimulation of IgA-producing B cells, thus aggravating the disease. The role of IL-10 in disease progression is further supported by the finding that compared to healthy donors, whole blood cultures from IgAN patients are more prone to produce IL-10 after stimulation with lipopolysaccharide or phytohemagglutinin [26].
2. **Tn syndrome:** Tn syndrome is characterized by Tn antigen expression on all major blood cell lineages. Patients with Tn syndrome are not clinically affected, except for minor signs of hemolysis or thrombocytopenia [27]. Tn syndrome is associated with a somatic mutation of COSMC, thereby preventing T-synthase function [28]. Interestingly, only 1–2% of T cells are affected. As binding of MGL to Tn antigen positive CD45 on T cells induces T cell apoptosis [29], it is tempting to speculate that Tn antigen positive T cells are cleared from the circulation and, thus, undetectable in these patients.
3. **Inflammatory bowel disease:** also in an inflammatory autoimmune setting *O*-glycans appear to be important for disease progression. Mice with an intestinal epithelial cell-specific loss of Core 1-derived *O*-glycans spontaneously develop colitis, suggesting a protective role of Core 1 in preventing intestinal inflammation [30]. Indeed, also patients with active ulcerative colitis show impaired expression of intestinal glycans and an accompanying increase in truncated glycans. An increased amount of the sTn antigen could be detected in 18% of the patients, compared to 2% in the control patients. Interestingly, the aberrant glycosylation profile was shown to be reversible upon remission and was significantly correlated to the extent of inflammation [31].

## 3. The Interplay between Tumor Cells and the Immune System

The tumor microenvironment is a complex and intricate network of tumor cells, stromal cells, and infiltrating immune cells. The immune system is able to control cancer development to a certain extent, since immunodeficient mice and immunocompromised humans, such as AIDS patients, develop tumors more readily [32,33]. These findings have led to the immunosurveillance theory [34,35], which assumes that the immune system constantly detects and eradicates evolving tumors, even before they become clinically visible. Nevertheless, immunosurveillance is not sufficient as malignant tumors do develop and, therefore, the updated concept of tumor immunoediting [36] is a more complete explanation on the role of immune cells in tumor progression. This concept conveys that tumor cells acquire the potency to evade ongoing immune responses by reducing immune recognition, by increasing their resistance against immune attack and by creating an immunosuppressive tumor microenvironment.

As the host’s immune system appears to be a crucial factor in tumor progression and metastasis formation, the immune status of a particular patient has become indispensable for predicting prognosis and response to therapy. This has led to the classification using the immunoscore, first introduced by Galon *et al.* [37]. The immunoscore is based on the immune infiltration in the tumor microenvironment and can be applied to identify high-risk patients who would benefit from immunotherapy. In a variety of solid tumors, high frequencies of tumor-infiltrating lymphocytes (TILs) were correlated with increased survival [38,39,40,41,42], whereby high frequencies of memory T cells are particularly associated with a lack of early metastatic invasion [43]. Moreover, in a longitudinal study, high cytotoxic activity of peripheral-blood lymphocytes is negatively correlated with cancer incidence [44]. Indeed, increased frequencies of anti-tumor cytotoxic CD8^+^ T cells (CTLs) at the center and the invasive margin of the tumor are positively correlated with increased survival [40,41]. Like CTLs, NK cells are able to lyse tumor cells, however the hypoxic microenvironment of the tumor reduces expression of the major activating NK-cell receptors, causing an impaired NK cell-mediated tumor kill [45]. Dendritic cells (DCs) capture, process, and (cross-) present antigens to naïve CD4^+^ and CD8^+^ T cells and are, therefore, the main instigators in initiating adaptive immunity. However, the number of DCs in the blood of breast, head and neck, and lung cancer patients are reduced and their maturation capacity is impaired compared to healthy blood DCs [46]. In agreement with this, tumor infiltration of mature DCs has been correlated with a better clinical outcome [47]. Tumor-associated macrophages (TAMs) can promote tumor progression by suppressing effector T cell responses through the production of anti-inflammatory cytokines such as IL-10 and TFGβ. Accordingly, TAM infiltration is also correlated with bad prognosis [48].

Key players in the suppression of anti-tumor immunity are the regulatory T cells (Tregs). Indeed, a low CTL/Treg ratio has been associated with poor clinical outcome in ovarian [49] and gastric cancer [50]. In addition, the tumor cells, themselves, contribute to immune suppression through the secretion of IL-10 and TGFβ and chemokines that recruit Tregs to the tumor site. Together, this creates the suppressive tumor microenvironment, preventing an effective tumor immune attack. The immune-related cancer evasion strategies were recently reviewed in more detail by others [47,51].

## 4. Immune Receptors Involved in the Recognition of Tumor-Associated *O*-glycans

The interaction between the immune system and the truncated *O*-glycans present on tumor cells is mediated by a diverse set of carbohydrate-binding receptors, commonly known as lectins. These glycan-binding receptors have been shown to be important immune regulators both in health and disease [52]. Especially, antigen presenting cells (APCs) express a wide variety of carbohydrate-binding receptors, including the C-type lectin receptors (CLRs) and the Sialic acid-binding immunoglobulin-type lectins (Siglecs). CLRs are able to internalize their ligands for processing and presentation to T cells. In addition, CLRs possess signaling properties to actively modify DC and macrophage function. The human CLR macrophage galactose-type lectin (MGL, CD301) recognizes terminal GalNAc moieties (Table 1), such as Tn and sTn antigen and is, therefore, a prime receptor for the aberrant *O*-glycans in cancer [53,54,55]. In mice, two MGL homologs with different binding specificities exist. Mouse MGL1 (mMGL1) primarily recognizes Lewis X, while mouse MGL2 (mMGL2) interacts with Tn antigen and, in contrast to human MGL, with T antigen and Core 2 structures [56] (Table 1). MGL is mainly expressed by immature and tolerogenic dendritic cells and macrophages [57], suggesting that MGL may play a role in immune regulation [58]. Moreover, mMGL1 and mMGL2 are markers for alternatively activated macrophages [59], while in humans high MGL expression is considered a marker for TAMs [60]. Research into the MGL expression on TAMs demonstrated that MGL levels on human TAMs are three times higher compared to *in vitro* generated macrophages [61]. An immunomodulatory role of MGL is further supported by the finding that high MGL binding in stage III colon cancer patients is associated with a poorer disease-free survival [62].

Ligands for MGL are mainly expressed on mucins produced by epithelial cancers. Healthy epithelial cell-derived mucins are characterized by an extended Core 2 glycan phenotype and they do not carry the cancerous truncated *O*-glycans. MGL is able to interact with various types of cancer-related-mucins from which two are extensively studied, namely mucin 1 (MUC1) and mucin 2 (MUC2). More importantly, APCs are able to distinguish between healthy and tumor-derived mucins through the MGL receptor [63,64,65]. The binding of MGL to Tn antigen on MUC1 appears to be independent of the sialylation status of Tn antigens, as MGL binds Tn-MUC1 and sTn-MUC1 with similar affinity [58].

Sialic acid-binding immunoglobulin-type lectins (Siglecs) specifically recognize sialic acids, which are generally the outermost sugars on a multitude of carbohydrate structures. Siglecs are widely expressed within the immune system and the majority of them contain an immunoreceptor tyrosine-based inhibitory motif (ITIM) in their cytoplasmic domain, again indicative of an immune regulatory function. In contrast to MGL, Siglecs recognize and bind normal self-antigens, which are usually decorated with sialic acids. Thus, the inhibitory function of Siglecs might be a way to tolerate the immune system to self-glycans. Sialylated glycans, therefore, have been proposed to act as self-associated molecular patterns (SAMPs) to maintain immune homeostasis and to dampen reactivity following an immune response [66]. Several pathogens, including *Neisseria gonorrhoeae* and group B *Streptococccus*, evade the immune system by decorating themselves with sialic acids, thus mimicking the host’s self-glycans. As CD33-related Siglecs can dampen hosts’ inflammatory immune responses, engagement of Siglec-5 and Siglec-9 by the bacterial sialic acids could, thus, be considered an immune manipulatory mechanism. It is, therefore, generally believed that Siglecs underwent rapidly evolutionary changes to combat with sialic acid-expressing pathogens [67].

In line with this, the hypersialylation, often observed on tumor cells might be one of the mechanisms by which tumors evade immune attack. However, which Siglecs are involved in the recognition of tumor-associated sTn and sT antigen and whether binding subsequently induces Siglec-mediated signaling is not extensively studied. To date, there are at least 13 different Siglecs described in humans and nine in mice. In contrast to the CD33-related Siglecs, Siglec-1 (also known as Sialoadhesion and CD169) has no ITIM motif and could, therefore, be an important antigen capture receptor to promote anti-tumor immunity. Indeed, selective targeting of Siglec-1^+^ macrophages induces an efficient CD8^+^ T cell response [68] and promotes germinal B-cell responses in mice [69]. Siglec-1 is also able to engage MUC1 from breast cancer cell lines in a sialic acid-dependent manner and Siglec-1^+^ macrophages are found in close contact with breast carcinoma cells [70]. However, whether Siglec-1-mediated signaling is promoting or inhibiting tumor progression is currently not known. Siglec-2 (also known as CD22) is expressed by B cells and has been shown to bind mucins derived from colon cancer cells. Upon mucin binding to CD22, the phosphorylation of ERK-1/2 was decreased, indicating a downregulation of B cell receptor signal transduction [71]. Additionally, Siglec-3 and Siglec-9 expressed by monocyte-derived DCs (moDCs) have been demonstrated to bind MUC2 via the sTn antigen epitope (Table 1), although the subsequent response is different. Binding of MUC2 to Siglec-3 induces apoptosis of moDCs, while binding to Siglec-9 on moDCs decreases IL-12 production, while leaving IL-10 production unaffected [72,73].

To summarize, immune cells can specifically recognize tumor-associated *O*-glycans through expression of CLRs, like MGL, and Siglecs. Based on their expression patterns and inhibitory signaling, these receptors have been implicated in the suppression of the anti-tumor immune response. Due to their extensive *O*-glycosylation, especially tumor-derived mucins carry a dense array of truncated *O*-glycan ligands for both the MGL and Siglec receptors.

## 5. Effect of Aberrantly-Glycosylated Mucins on Immunity

The high molecular weight mucin proteins are the most abundant carriers of *O*-glycans. They are produced by epithelial cells and from the at least 20 different mucin types known, especially MUC1 and MUC2 are well studied in regard to their immunomodulatory properties. Mucins consist of 5–500 tandem-repeat domains enriched in serine, proline, and threonine amino residues. Each tandem repeat contains at least 5–100 potential glycosylation sites, resulting in a heavily *O*-glycosylated mucin protein [74]. Mucins produced by healthy cells mainly contain extended Core 2-based complex glycans, while the tumor-derived mucins mainly express truncated and immature *O*-glycans [75].

### 5.1. Uptake and Processing of Mucins by DCs

The main immunological function of DCs is to instruct and activate naïve CD8^+^ and CD4^+^ T cells through the presentation of immunogenic peptides in MHC class I and II molecules, respectively. The glycosylation status of a peptide does not necessarily interfere with antigen presentation as both MHC class I and II molecules are able to bind and present glycosylated peptides [76,77]. Synthetic Tn antigen-containing MUC1 glycopeptides, representing the tumor-associated MUC1, are processed to smaller fragments for loading into the MHC class II molecule without removing the glycans attached to the original MUC1 peptide [78]. Strikingly, the degradation of MUC1 glycopeptides is not affected by the length of the glycan [78] but the peptide cleavage sites are qualitatively and quantitatively influenced by *O*-gycosylation for presentation in both MHC class I [79] and MHC class II [80]. Unlike the presentation of MUC1 glycopeptides in MHC class II, published data on presentation in MHC class I are contradictory. Tn-MUC1 glycopeptides derived from CHO-transfected cell lines [64], as well as MUC1 glycopeptides from pooled ascites fluid from patients with metastatic breast and pancreatic cancer [81], are processed and presented in both MHC class I and II molecules in DCs, supporting the ability of DCs to specifically initiate adaptive immunity to tumor-associated mucins. Nevertheless, Madsen *et al.* demonstrated that Tn glycosylation of an ovalbumin (OVA)-MUC1 fusion peptide inhibited the presentation of the fusion peptides by MHC class I and abolished MUC1-specific CD8^+^ T cell responses. The same fusion peptide did, however, promote presentation by MHC class II and elicited a specific antibody response [82]. Since Tn-OVA conjugates are able to induce increased CD8^+^ T cell proliferation compared to the unconjugated OVA [83], the observed contradiction is likely not due to the use of OVA as a backbone in the OVA-MUC1 fusion construct. Since the degradation of glycopeptides depends on the attachment site of the glycans, glycosylation might also affect the cross-presentation pathway of DCs and consequently presentation in the MHC class I molecule, thus providing an explanation for the observed contradictory results.

As tumor cells express and, in case of MUC2, secrete mucins, DCs are more likely to encounter whole mucin proteins instead of mucin glycopeptides. DCs are equally capable of endocytosing MUC1 glycoproteins, but in contrast to MUC1 glycopeptides, the MUC1 glycoproteins are not transported to late endosomes or MHC class II loading compartments for processing and binding to the MHC class II molecule [81]. It has been postulated that abundant mannose structures present on MUC1 glycoproteins bind the mannose receptor and prevent dissociation of MUC1 in the early endosomes, thus leading to entrapment of MUC1 in this compartment [81]. In contrast, Tn antigen-containing MUC1 is internalized through MGL and subsequently accumulates in MHC class II loading compartments [64], supporting the idea that the addition of Tn antigen averts binding to mannose receptors and thereby entrapment in the endosome. Co-localization of the Tn-MUC1 glycoprotein with MHC class I is not observed [64]; hence, it is unlikely that DCs are able to process and present Tn-MUC1 glycoproteins in MHC class I molecules. Clearly, mucin glycoproteins undergo a different intracellular routing in DCs than mucin glycopeptides, and depending on their glycosylation pattern the routing of mucin glycoproteins is re-adjusted to different intracellular compartments.

### 5.2. Influence of Mucin Engagement on Adaptive Immunity

The maturation status of the DCs is crucial for the subsequent naïve T cell differentiation and involves expression of co-stimulatory and inhibitory molecules and production of pro- or anti-inflammatory cytokines. DCs are thus able to master the balance between inflammatory responses and immune suppression. The influence of mucin-type proteins on DC maturation has been primarily studied using co-culture systems. For instance, MUC2-stimulated monocyte-derived DCs decrease their secretion of the IL-12 cytokine in a glycosylation-dependent manner [73]. In DCs cultured with recombinant sT-MUC1, the expression of several co-stimulatory molecules, such as CD86 and CD40 and antigen presenting molecules CD1d and HLA-DR was decreased. In contrast, CD1a and mannose receptor levels were increased. The cytokine profile of sT-MUC1-stimulated DCs is characterized as IL-10^high^ and IL-12^low^ [84,85], suggesting that sT-MUC1 prevents DC maturation and induces a regulatory DC phenotype. Indeed, the sT-MUC1-stimulated DCs were defective in triggering pro-inflammatory immune responses in both allogeneic and autologous settings [85]. Together these studies show that engagement of tumor-associated mucins by DCs prevents effective induction of subsequent immune responses, pointing to a suppressive action of mucins on adaptive immunity through the induction of tolerogenic DCs.

T cells and B cells are the two main players in adaptive immunity. Natural antibodies, produced by B cells, are present in the body without prior external immune activation. Such natural antibodies directed against MUC1 glycopeptides can be detected in the serum of breast cancer patients. Interestingly, these natural antibodies have a binding preference for glycosylated MUC1 peptides compared to the unglycosylated MUC1 [86]. Antibodies play a crucial role in suppressing tumor growth by binding tumor cells, thereby facilitating recognition and destruction by the immune system via antibody-dependent cell-mediated cytotoxicity. In breast cancer patients, a strong anti-sT-MUC1 antibody response was associated with reduced rate and a delay in metastases formation [87], suggesting that antibodies could indeed play a role in dampening breast cancer progression. As plasma B cells are the sole producers of antibodies, the effect of mucins on B cells was investigated in *in vivo* tumor models. Mice bearing a mucin-producing mammary tumor had a reduced number of splenic B cells compared to mice bearing non-producing tumors. This effect was probably due to the presence of mucins in the bloodstream which can engage CD22 on splenic B cells, thereby downregulating B cell receptor signal transduction, as discussed previously [71].

Although efficient CTL cytotoxicity is crucial for tumor immune attack, not much is known about the direct effect of mucins on the CD8^+^ T cell responses. This may be explained by the finding that DCs are not able to efficiently process and present mucin glycoproteins in the MHC class I molecule.

## 6. Effect of Tumor-Associated *O*-glycans on APCs and the Initiation of Adaptive Immunity

Since truncated *O*-glycans are specifically recognized by lectins, such as MGL and the Siglecs, the immunomodulatory capacity of these receptors has been addressed by targeting these receptors through antibodies or *O*-glycans coupled to model tumor antigens. Additionally, knockdown of glycosyltransferases in *in vivo* tumor models has been used to investigate the effect of tumor glycosylation on the immune system. The results of these studies are described below.

### 6.1. Immune Modulation by MGL^+^ APCs

Immature DCs continuously sample their environment for invading pathogens. Upon pathogen encounter, DCs mature and migrate to the lymph node to initiate adaptive immunity by activating naïve T cells. The C-type lectin MGL is only expressed on immature DCs, where it hampers the DC migratory response [53]. Tumor cells might take advantage of this by expressing high levels of MGL ligands that, in turn, prevent the migration of the tumor-infiltrating DCs to the lymph nodes and, thus, prevent initiation of anti-tumor adaptive immune responses. Normally, mature DCs in the lymph node are able to skew naïve CD4^+^ T cells towards distinct T helper cell populations, such as T helper 1 and 2 cells. The differentiation of the T helper cell subsets is amongst others dependent on the DCs subset. Murine dermal DCs (dDCs) specifically express the mMGL2 lectin [88]. In contrast to human MGL^+^ DCs, the migratory capacity of these mMGL2^+^ dDCs is not affected, as mMGL2^+^ dDCs are capable of transporting protein antigens, injected in the footpad, efficiently to the skin-draining lymph nodes. Moreover, the percentage of mMGL2^+^ dDCs in the skin-draining lymph node increases dramatically when papain is used as an adjuvant. Papain is a potent T helper 2-inducing adjuvant, indicating that mMGL2^+^ dDCs are required for the development of antigen-specific T helper 2 responses. Nevertheless, mMGL2^+^ dDCs are not required for the differentiation of T follicular helper cells or germinal center responses induced by pathogens, such as *Nippostrongylus brasiliensis* [88]*.* However, Freire *et al.* observed a strong germinal B cell reaction after intradermal immunization of Tn-dendrimers in mice, resulting in anti-Tn antibodies [89]. Since Tn antigen is a ligand for mMGL2, Tn antigen might play a role in the development of T helper 2 responses including a strong B cell activation. The role of Tn antigen on T helper 2 responses in mice is further supported by a study of Chen *et al.* When the T-synthase enzyme was knocked down in the murine posterior tibialis muscle, leading to high levels of Tn antigen, the splenic CD4^+^ T cells showed an enhanced production of T helper 2 cytokines, namely IL-10 and IL-4, after transplantation of skin grafts [90]. However, whether the observed effects were mMGL2 mediated or not was not addressed. Together these results suggest that Tn antigen directs the activation of T helper 2 responses through mMGL2 in mice. Whether immune responses directed to Tn antigen positive tumors are more prone to generate detrimental T helper 2-type reactions and, thereby, indirectly prevent the preferential anti-tumor T helper 1 immunity, is, as far we know, not described in literature.

The DC maturation program is initiated after pathogen binding to specific pattern recognition receptors, such as the Toll-like receptors (TLRs), on the DC. In addition, TLRs can recognize specific danger molecules released or expressed by tumor cells. CLR-glycan interactions have already been shown to modify TLR-mediated signaling, leading to a fine-tuning of the pathogen-specific immune responses [91]. The MGL-Tn/sTn interaction could, therefore, also potentially modify the DCs’ ability to respond to danger signals originating from the tumor. Indeed, upon MGL engagement, DCs enhance their TLR-2-mediated signaling resulting in an increased secretion of the anti-inflammatory cytokine IL-10 and a tolerogenic DC phenotype [92]. Furthermore, these MGL-licensed DCs are able to specifically promote the differentiation of regulatory T cells [93]. The tolerogenic nature of the human MGL^+^ DCs is even further supported by a study investigating the interaction between DCs and T cells. Interestingly, effector T cells carry MGL ligands on their CD45 molecules. Binding of MGL to CD45 on effector T cells induces T cell apoptosis, which is dependent on concomitant T cell receptor triggering [29]. As highly activated effector T cells increase their Tn antigen expression [92], MGL^+^ APCs in the tumor microenvironment could be crucial in dampening cytotoxic reactions by inducing apoptosis of tumor-infiltrating CD8^+^ T cells.

As discussed earlier, mouse and human MGL appear to be quite different regarding their immunological function. Singh *et al.* observed enhanced antigen-specific CD4^+^ and CD8^+^ T cell responses with the use of OVA coupled to Tn antigen, compared to unmodified OVA proteins. Here, the effects were mMGL2-mediated, as Tn-OVA conjugates were endocytosed and presented in MHC class I and II molecules in a mMGL2-dependent manner [83]. For both human and mice, targeting of MGL with an antibody-conjugate has been shown to increase antigen presentation by DCs [89,94], indicating that the observed differences are not due to inefficient antigen presentation.

**Figure 2:**
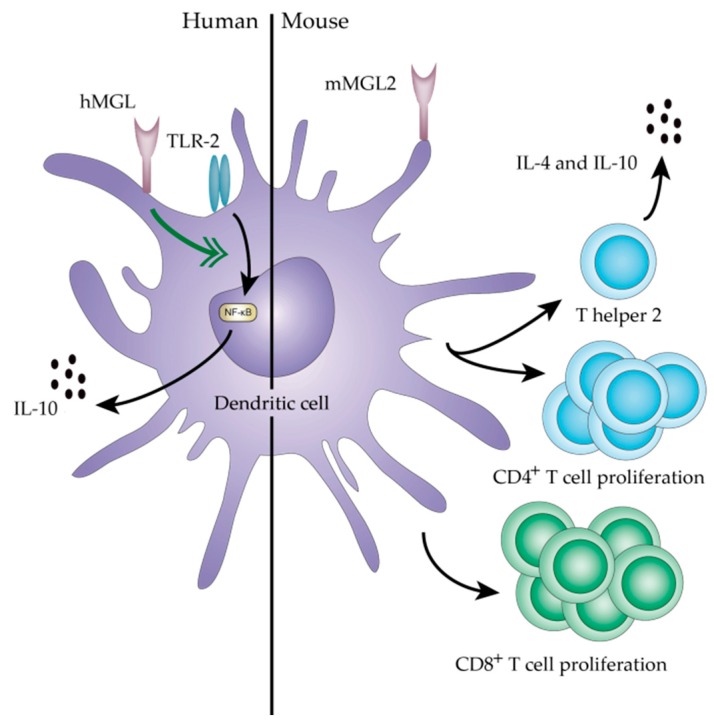
Immune modulation by human and mouse MGL^+^ dendritic cells. Depicted are the MGL-mediated effects on DC and T cell responses after MGL engagement by tumor-associated glycoproteins and *O*-glycans.

In conclusion, mMGL2^+^ dDCs play a role in T helper 2 immune responses and targeting of mMGL2 with Tn-OVA glycoconjugates induces enhanced CD4^+^ and CD8^+^ T cell responses (Figure 2). In contrast, human MGL^+^ DCs are hampered in their migratory capacity and adopt a tolerogenic phenotype upon engagement of MGL. That human MGL appears to have an immune-suppressive action through the specific induction of effector T cell apoptosis may provide an explanation for the correlation between MGL binding and reduced survival in colon cancer patients [62].

As depicted in Figure 1, truncated *O*-glycans can be sialylated and, thereby, form sTn and sT antigen structures. Sialylated glycan structures can be recognized by Siglec receptors which, through their intracellular ITIM motifs, have the potential to dampen anti-tumor immunity. To investigate the role of sTn on immune modulation, human moDCs were co-cultured with a bladder cancer cell line (the MCR cell line) that was transduced with the ST6GalNAc-I enzyme to induce sTn expression (MCR-sTn). MoDCs co-cultured with MCR-sTn cells had an immature phenotype characterized by lower expression of MHC class II, CD80, and CD86 compared to the moDCs co-cultured with control MCR cells. Interestingly, the moDCs from the MCR-sTn co-culture were unresponsive to further maturation stimuli and produced only low levels of IL-12 and TNFα [95]. Both the reduction in maturation markers and the low levels of pro-inflammatory cytokines suggests the generation of tolerogenic DCs upon contact with sTn-expressing cells. This is further supported by the observation that MCR-sTn-stimulated moDCs were not able to activate T cells as measured by the expression of the early T cell activation marker CD69. Furthermore, these T cells displayed a Foxp3^high^, IFNγ^low^ phenotype, representing a typical regulatory T cell profile [95]. Importantly, similar results were obtained using a sTn positive breast cancer cell line, arguing that the sTn antigen effect on DC immunity is irrespective of the cancer type. Blockade of CD44 and MUC1 was able to prevent the tolerance-inducing effect on DCs, indicating a pivotal role of CD44 and MUC1 in the observed immune modulatory effects by sTn antigen [95].

### 6.2. Sialylated O-glycans and Immunity

The Treg-promoting capacity of sialic acids is further exemplified in the mouse melanoma (B16) cancer model. Isogenic B16 cell lines with either high or low expression of sialic acids were generated through knockdown of the sialic acid transporter SLC35A1. *In vivo* the growth of the B16 sialic acid low tumors was significantly decreased compared to B16 sialic acid high tumors. More importantly, in the B16 sialic acid low tumors more effector T cells and a diminished number of regulatory T cells were detected at the tumor site [96]. Intriguingly and similar to DCs co-cultured with sTn antigen-positive bladder cancer cells [95], DCs primed with sialic acid-OVA conjugates skewed naïve T cells towards antigen-specific Foxp3^high^ IFNy^low^ regulatory T cells in a Siglec-E-dependent manner [97]. As Siglec-E is not only expressed by DCs, but also on mouse neutrophils, monocytes, and macrophages, the sialic acid-Siglec-E interaction might modify additional immune-related pathways.

As discussed earlier, sTn present on MUC2 is recognized by human Siglec-3 and Siglec-9 expressed by moDCs and failed to induce high production of IL-12 [72,73]. Triggering of the Siglec-9 receptor on macrophages increased IL-10 and decreased TNFα production after stimulation with the TLR ligand lipopolysaccharide or peptidoglycan [98]. Another sTn-binding Siglec is Siglec-15, which is expressed by macrophages and DCs and, interestingly, also found on TAMs in various human carcinoma tissues including lung, rectal, and hepatocellular carcinoma. Engagement of Siglec-15 on macrophages with sTn augments the TGFβ production by these cells [99], suggesting that Siglec-15 on macrophages may contribute to tumor progression by TGFβ-mediated immune modulation.

**Figure 3:**
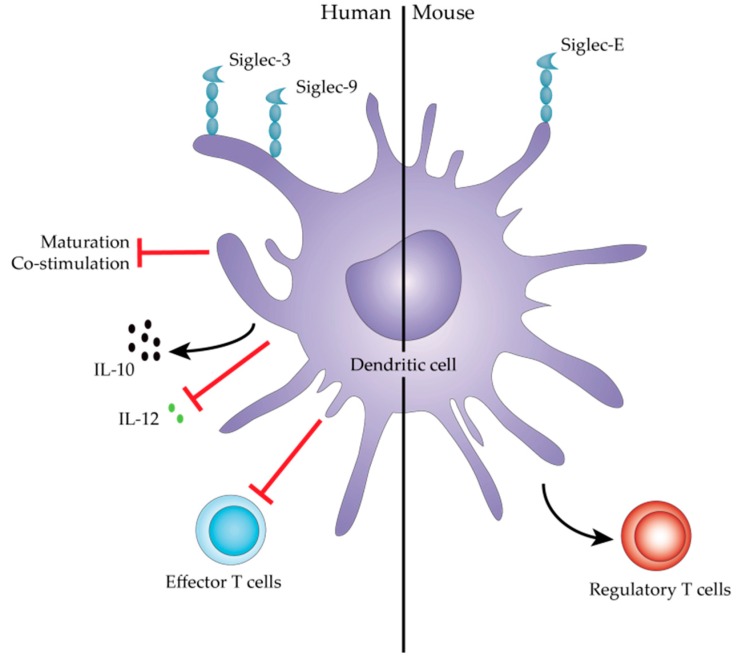
Immune modulation by human and mouse Siglec^+^ dendritic cells. Depicted are the Siglec-mediated effects on DC and T cell responses after Siglec engagement by tumor-associated glycoproteins and sialylated *O*-glycans, or through co-culture with sialylated tumor cells. In mice, these effects are mediated by Siglec-E, whereas in humans Siglec-3 and -9 are known to modulate DC immunity.

Overall, a concept emerges in which sialic acids present on tumor cells play a central role in immune suppression (Figure 3) and, thereby, tumor immune evasion, likely through their interaction with inhibitory Siglec receptors. Remarkably, no research is dedicated to the role of sialyl-T and di-sialyl T antigen on immune modulation, while the unsialylated T antigen and the responsible glycosyltransferases T-synthase and COSMC are well studied. It is highly probable that the sialylated T antigens are also recognized by certain Siglec receptors on immune cells. However, whether this interaction contributes to tumor immune evasion is subject for future studies.

## 7. Effect of Tumor-Associated *O*-glycans on Natural Killer Cells

In addition to DCs and macrophages, not much is known about the effect of *O*-glycans on the other innate immune cell subsets. Natural killer cells (NK cells), as the name already implies, are able to eliminate infected, as well as damaged and malignant, cells very efficiently. Their cytotoxic action is balanced by signaling through activating and inhibitory receptors on the NK cell surface. Already in the 1980s, NK cells were first implicated in tumor immunosurveillance, when researchers observed a higher incidence of defective NK cell function in individuals with cancer [100,101]. Early experiments using co-cultures of blood mononucluear cells, containing NK cells and K562 tumor cells, a classical NK target, revealed that NK cells were less able to kill tumor cells in the presence of sialic acids, while other glycans, such as mannose and galactose, had no effect [102]. Although not a direct proof, it does suggest that sialic acids participate in preventing NK cytotoxicity. More specifically, mucins derived from sheep, bovine, and porcine submaxillary glands that carry sTn are able to inhibit NK-mediated tumor cell killing, while mucins derived from human breast and lung cancer cells that lack sTn antigen have no effect. Together, these results suggest that sTn antigen can directly modulate NK cell function [103].

A more recent study revealed that desialylated tumor cells (from a mouse 3-methylcholanthrene-induced fibrosarcoma) induce higher secretion of IFNγ by NK cells, indicative of a more active NK response [104]. Moreover, the NK-activating receptor NKG2D was shown to be responsible for the interaction between NK cells and the desialylated ligands on the tumor cells. *In vivo*, desialylated tumor cells show a reduced tumor growth compared with to fully sialylated wild-type tumor cells [104], which is also a NK-mediated phenomenon [96].

The interaction between sialylated tumor cells and NK cells occurs via the inhibitory Siglec-7 and Siglec-9 receptors on the human NK cell surface. Ligands for Siglec-7 and Siglec-9, which are distinct sialoglycan determinants such as ganglioside GD3, are broadly expressed on different human cancer types and protect the cancer cells from NK cell-mediated cytotoxicity [105]. Moreover, increasing the sialylation status of multiple cancer lines decreases their susceptibility to antibody-dependent NK cytotoxicity, through the recruitment of Siglec-7 [106].

Although commonly accepted that NK cells are instrumental in eliminating tumor cells, the effect of tumor cell glycosylation on NK cell function has not been extensively studied and forms material for future research.

## 8. Conclusions

Avoiding immune destruction is one of the hallmarks of cancer. We postulate that the aberrant glycosylation of tumors is one of the major factors underlying the development of an immune suppressive tumor microenvironment. Tumor-associated *O*-glycans induce a tolerogenic programming of tumor-resident DCs, characterized with low expression of co-stimulatory molecules, high IL-10 secretion and an incapacity to induce of effector T cell responses. Engagement of truncated *O*-glycans by lectin receptors on tumor-associated macrophages likewise promotes production of anti-inflammatory cytokines, such as IL-10 and TGFβ. Cytotoxic NK cells are crucial in eliminating tumor cells, but surprisingly, the role of tumor-associated *O*-glycans on the function of NK cells is scarce. Clearly, more research is needed to fully understand the impact of tumor glycans on anti-tumor immunity. Some of the outstanding questions that await answering, include: clarification of the specific contribution of each aberrant *O*-glycan structure (Tn, sTn, T, and sT), including the importance of the peptide/protein backbone, to tumor immune evasion, the cells and receptors involved and elucidation of the molecular mechanisms by which tumor cells employ glycans to avoid anti-tumor immunity. The impact of the tumor type may be one of the confounding factors and should be taken into consideration when addressing these issues. Increasing our understanding of tumor cell glycosylation and its regulation may create novel strategies to alleviate tumor-mediated immune suppression by directly interfering with the tumor glycome and its interaction with antigen presenting cells.

## Figures and Tables

**Table 1:** Immune receptors involved in the recognition of tumor-associated *O*-glycans.

Species	Receptor	*O*-glycan structure	
human	hMGL	Tn antigen	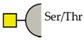
		Sialyl-Tn antigen	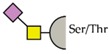
mouse	mMGL1	Lewis X	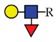
	mMGL2	Tn antigen	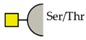
		T antigen	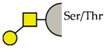
		Core-2	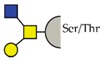
human	hSiglec-3	Sialyl-Tn antigen	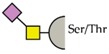
	hSiglec-9	Sialyl-Tn antigen	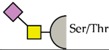

## References

[1] Hoseki J., Ushioda R., Nagata K. (2010). Mechanism and components of endoplasmic reticulum-associated degradation. J. Biochem..

[2] Pinho S.S., Reis C.A. (2015). Glycosylation in cancer: Mechanisms and clinical implications. Nat. Rev. Cancer.

[3] Brockhausen I., Yang J., Lehotay M., Ogata S., Itzkowitz S. (2001). Pathways of mucin *O*-glycosylation in normal and malignant rat colonic epithelial cells reveal a mechanism for cancer-associated Sialyl-Tn antigen expression. Biol. Chem..

[4] Marcos N.T., Pinho S., Grandela C., Cruz A., Samyn-Petit B., Harduin-Lepers A., Almeida R., Silva F., Morais V., Costa J. (2004). Role of the human ST6GalNAc-I and ST6GalNAc-II in the synthesis of the cancer-associated sialyl-Tn antigen. Cancer Res..

[5] Gill D.J., Tham K.M., Chia J., Wang S.C., Steentoft C., Clausen H., Bard-Chapeau E.A., Bard F.A. (2013). Initiation of GalNAc-type *O*-glycosylation in the endoplasmic reticulum promotes cancer cell invasiveness. Proc. Natl. Acad. Sci. USA.

[6] Wang Y., Ju T., Ding X., Xia B., Wang W., Xia L., He M., Cummings R.D. (2010). Cosmc is an essential chaperone for correct protein O-glycosylation. Proc. Natl. Acad. Sci. USA.

[7] Ju T., Cummings R.D., Canfield W.M. (2002). Purification, characterization, and subunit structure of rat core 1 Beta1,3-galactosyltransferase. J. Biol. Chem..

[8] Song K., Herzog B.H., Fu J., Sheng M., Bergstrom K., McDaniel J.M., Kondo Y., McGee S., Cai X., Li P. (2015). Loss of Core 1-derived O-Glycans Decreases Breast Cancer Development in Mice. J. Biol. Chem..

[9] Wu Y.M., Liu C.H., Huang M.J., Lai H.S., Lee P.H., Hu R.H., Huang M.C. (2013). C1GALT1 enhances proliferation of hepatocellular carcinoma cells via modulating MET glycosylation and dimerization. Cancer Res..

[10] Radhakrishnan P., Dabelsteen S., Madsen F.B., Francavilla C., Kopp K.L., Steentoft C., Vakhrushev S.Y., Olsen J.V., Hansen L., Bennett E.P. (2014). Immature truncated *O*-glycophenotype of cancer directly induces oncogenic features. Proc. Natl. Acad. Sci. USA.

[11] Cao Y., Karsten U.R., Liebrich W., Haensch W., Springer G.F., Schlag P.M. (1995). Expression of Thomsen-Friedenreich-related antigens in primary and metastatic colorectal carcinomas. A reevaluation. Cancer.

[12] Ghazizadeh M., Ogawa H., Sasaki Y., Araki T., Aihara K. (1997). Mucin carbohydrate antigens (T, Tn, and sialyl-Tn) in human ovarian carcinomas: Relationship with histopathology and prognosis. Hum. Pathol..

[13] Imai J., Ghazizadeh M., Naito Z., Asano G. (2001). Immunohistochemical expression of T, Tn and sialyl-Tn antigens and clinical outcome in human breast carcinoma. Anticancer Res..

[14] Karsten U., Goletz S. (2013). What makes cancer stem cell markers different?. Springerplus.

[15] Pinho S.S., Carvalho S., Marcos-Pinto R., Magalhaes A., Oliveira C., Gu J., Dinis-Ribeiro M., Carneiro F., Seruca R., Reis C.A. (2013). Gastric cancer: Adding glycosylation to the equation. Trends Mol. Med..

[16] Marcos N.T., Bennett E.P., Gomes J., Magalhaes A., Gomes C., David L., Dar I., Jeanneau C., DeFrees S., Krustrup D. (2011). ST6GalNAc-I controls expression of sialyl-Tn antigen in gastrointestinal tissues. Front. Biosci. (Elite Ed).

[17] Ferreira J.A., Videira P.A., Lima L., Pereira S., Silva M., Carrascal M., Severino P.F., Fernandes E., Almeida A., Costa C. (2013). Overexpression of tumour-associated carbohydrate antigen sialyl-Tn in advanced bladder tumours. Mol. Oncol..

[18] Laack E., Nikbakht H., Peters A., Kugler C., Jasiewicz Y., Edler L., Hossfeld D.K., Schumacher U. (2002). Lectin histochemistry of resected adenocarcinoma of the lung: Helix pomatia agglutinin binding is an independent prognostic factor. Am. J. Pathol..

[19] Chia J., Goh G., Bard F. (2016). Short *O*-GalNAc glycans: Regulation and role in tumor development and clinical perspectives. Biochim. Biophys. Acta.

[20] Brockhausen I. (2006). Mucin-type *O*-glycans in human colon and breast cancer: Glycodynamics and functions. EMBO Rep..

[21] Karsten U., Goletz S. (2015). What controls the expression of the core-1 (Thomsen-Friedenreich) glycotope on tumor cells?. Biochemistry (Mosc).

[22] Allen A.C., Bailey E.M., Barratt J., Buck K.S., Feehally J. (1999). Analysis of IgA1 *O*-glycans in IgA nephropathy by fluorophore-assisted carbohydrate electrophoresis. J. Am. Soc. Nephrol..

[23] Hiki Y., Odani H., Takahashi M., Yasuda Y., Nishimoto A., Iwase H., Shinzato T., Kobayashi Y., Maeda K. (2001). Mass spectrometry proves under-O-glycosylation of glomerular IgA1 in IgA nephropathy. Kidney Int..

[24] Tomana M., Novak J., Julian B.A., Matousovic K., Konecny K., Mestecky J. (1999). Circulating immune complexes in IgA nephropathy consist of IgA1 with galactose-deficient hinge region and antiglycan antibodies. J. Clin. Investig..

[25] Van Vliet S.J., Bay S., Vuist I.M., Kalay H., Garcia-Vallejo J.J., Leclerc C., van Kooyk Y. (2013). MGL signaling augments TLR2-mediated responses for enhanced IL-10 and TNF-alpha secretion. J. Leukoc. Biol..

[26] De Fijter J.W., Daha M.R., Schroeijers W.E., van Es L.A., Van Kooten C. (1998). Increased IL-10 production by stimulated whole blood cultures in primary IgA nephropathy. Clin. Exp. Immunol..

[27] Berger E.G. (1999). Tn-syndrome. Biochim. Biophys. Acta.

[28] Ju T., Cummings R.D. (2005). Protein glycosylation: Chaperone mutation in Tn syndrome. Nature.

[29] Van Vliet S.J., Gringhuis S.I., Geijtenbeek T.B., van Kooyk Y. (2006). Regulation of effector T cells by antigen-presenting cells via interaction of the C-type lectin MGL with CD45. Nat. Immunol..

[30] Fu J., Wei B., Wen T., Johansson M.E., Liu X., Bradford E., Thomsson K.A., McGee S., Mansour L., Tong M. (2011). Loss of intestinal core 1-derived *O*-glycans causes spontaneous colitis in mice. J. Clin. Investig..

[31] Larsson J.M., Karlsson H., Crespo J.G., Johansson M.E., Eklund L., Sjovall H., Hansson G.C. (2011). Altered O-glycosylation profile of MUC2 mucin occurs in active ulcerative colitis and is associated with increased inflammation. Inflamm. Bowel. Dis..

[32] Goedert J.J., Cote T.R., Virgo P., Scoppa S.M., Kingma D.W., Gail M.H., Jaffe E.S., Biggar R.J. (1998). Spectrum of AIDS-associated malignant disorders. Lancet.

[33] Kaplan D.H., Shankaran V., Dighe A.S., Stockert E., Aguet M., Old L.J., Schreiber R.D. (1998). Demonstration of an interferon gamma-dependent tumor surveillance system in immunocompetent mice. Proc. Natl. Acad. Sci. USA.

[34] Burnet F.M. (1970). The concept of immunological surveillance. Prog. Exp. Tumor Res..

[35] Burnet M. (1964). Immunological Factors in the Process of Carcinogenesis. Br. Med. Bull..

[36] Dunn G.P., Bruce A.T., Ikeda H., Old L.J., Schreiber R.D. (2002). Cancer immunoediting: From immunosurveillance to tumor escape. Nat. Immunol..

[37] Galon J., Pages F., Marincola F.M., Thurin M., Trinchieri G., Fox B.A., Gajewski T.F., Ascierto P.A. (2012). The immune score as a new possible approach for the classification of cancer. J. Transl. Med..

[38] Zhang Z.Y., Zang R.Y., Tang M.Q., Chen J. (2003). Significance of systematic retroperitoneal lymphadenectomy at second-look laparotomy for ovarian cancer. Zhonghua Fu Chan Ke Za Zhi.

[39] Marrogi A.J., Munshi A., Merogi A.J., Ohadike Y., El-Habashi A., Marrogi O.L., Freeman S.M. (1997). Study of tumor infiltrating lymphocytes and transforming growth factor-beta as prognostic factors in breast carcinoma. Int. J. Cancer.

[40] Naito Y., Saito K., Shiiba K., Ohuchi A., Saigenji K., Nagura H., Ohtani H. (1998). CD8^+^ T cells infiltrated within cancer cell nests as a prognostic factor in human colorectal cancer. Cancer Res..

[41] Nakano O., Sato M., Naito Y., Suzuki K., Orikasa S., Aizawa M., Suzuki Y., Shintaku I., Nagura H., Ohtani H. (2001). Proliferative activity of intratumoral CD8^+^ T-lymphocytes as a prognostic factor in human renal cell carcinoma: clinicopathologic demonstration of antitumor immunity. Cancer Res..

[42] Schumacher K., Haensch W., Roefzaad C., Schlag P.M. (2001). Prognostic significance of activated CD8^+^ T cell infiltrations within esophageal carcinomas. Cancer Res..

[43] Pages F., Berger A., Camus M., Sanchez-Cabo F., Costes A., Molidor R., Mlecnik B., Kirilovsky A., Nilsson M., Damotte D. (2005). Effector memory T cells, early metastasis, and survival in colorectal cancer. N. Engl. J. Med..

[44] Imai K., Matsuyama S., Miyake S., Suga K., Nakachi K. (2000). Natural cytotoxic activity of peripheral-blood lymphocytes and cancer incidence: An 11-year follow-up study of a general population. Lancet.

[45] Balsamo M., Manzini C., Pietra G., Raggi F., Blengio F., Mingari M.C., Varesio L., Moretta L., Bosco M.C., Vitale M. (2013). Hypoxia downregulates the expression of activating receptors involved in NK-cell-mediated target cell killing without affecting ADCC. Eur. J. Immunol..

[46] Almand B., Resser J.R., Lindman B., Nadaf S., Clark J.I., Kwon E.D., Carbone D.P., Gabrilovich D.I. (2000). Clinical significance of defective dendritic cell differentiation in cancer. Clin. Cancer Res..

[47] Muenst S., Laubli H., Soysal S.D., Zippelius A., Tzankov A., Hoeller S. (2016). The immune system and cancer evasion strategies: Therapeutic concepts. J. Intern Med..

[48] Nabeshima A., Matsumoto Y., Fukushi J., Iura K., Matsunobu T., Endo M., Fujiwara T., Iida K., Fujiwara Y., Hatano M. (2015). Tumour-associated macrophages correlate with poor prognosis in myxoid liposarcoma and promote cell motility and invasion via the HB-EGF-EGFR-PI3K/Akt pathways. Br. J. Cancer.

[49] Preston C.C., Maurer M.J., Oberg A.L., Visscher D.W., Kalli K.R., Hartmann L.C., Goode E.L., Knutson K.L. (2013). The ratios of CD8^+^ T cells to CD4^+^ CD25^+^ FOXP3^+^ and FOXP3^−^ T cells correlate with poor clinical outcome in human serous ovarian cancer. PLoS ONE.

[50] Shen Z., Zhou S., Wang Y., Li R.L., Zhong C., Liang C., Sun Y. (2010). Higher intratumoral infiltrated Foxp3^+^ Treg numbers and Foxp3^+^/CD8^+^ ratio are associated with adverse prognosis in resectable gastric cancer. J. Cancer Res. Clin. Oncol..

[51] Joyce J.A., Fearon D.T. (2015). T cell exclusion, immune privilege, and the tumor microenvironment. Science.

[52] Ohtsubo K., Marth J.D. (2006). Glycosylation in cellular mechanisms of health and disease. Cell.

[53] Van Vliet S.J., Paessens L.C., Broks-van den Berg V.C., Geijtenbeek T.B., van Kooyk Y. (2008). The C-type lectin macrophage galactose-type lectin impedes migration of immature APCs. J. Immunol..

[54] Jegouzo S.A., Quintero-Martinez A., Ouyang X., dos Santos A., Taylor M.E., Drickamer K. (2013). Organization of the extracellular portion of the macrophage galactose receptor: A trimeric cluster of simple binding sites for N-acetylgalactosamine. Glycobiology.

[55] Mortezai N., Behnken H.N., Kurze A.K., Ludewig P., Buck F., Meyer B., Wagener C. (2013). Tumor-associated Neu5Ac-Tn and Neu5Gc-Tn antigens bind to C-type lectin CLEC10A (CD301, MGL). Glycobiology.

[56] Singh S.K., Streng-Ouwehand I., Litjens M., Weelij D.R., Garcia-Vallejo J.J., van Vliet S.J., Saeland E., van Kooyk Y. (2009). Characterization of murine MGL1 and MGL2 C-type lectins: Distinct glycan specificities and tumor binding properties. Mol. Immunol..

[57] Van Vliet S.J., van Liempt E., Geijtenbeek T.B., van Kooyk Y. (2006). Differential regulation of C-type lectin expression on tolerogenic dendritic cell subsets. Immunobiology.

[58] Beatson R., Maurstad G., Picco G., Arulappu A., Coleman J., Wandell H.H., Clausen H., Mandel U., Taylor-Papadimitriou J., Sletmoen M. (2015). The Breast Cancer-Associated Glycoforms of MUC1, MUC1-Tn and sialyl-Tn, Are Expressed in COSMC Wild-Type Cells and Bind the C-Type Lectin MGL. PLoS ONE.

[59] Raes G., Brys L., Dahal B.K., Brandt J., Grooten J., Brombacher F., Vanham G., Noel W., Bogaert P., Boonefaes T. (2005). Macrophage galactose-type C-type lectins as novel markers for alternatively activated macrophages elicited by parasitic infections and allergic airway inflammation. J. Leukoc. Biol..

[60] Solinas G., Schiarea S., Liguori M., Fabbri M., Pesce S., Zammataro L., Pasqualini F., Nebuloni M., Chiabrando C., Mantovani A. (2010). Tumor-conditioned macrophages secrete migration-stimulating factor: A new marker for M2-polarization, influencing tumor cell motility. J. Immunol..

[61] Allavena P., Chieppa M., Bianchi G., Solinas G., Fabbri M., Laskarin G., Mantovani A. (2010). Engagement of the mannose receptor by tumoral mucins activates an immune suppressive phenotype in human tumor-associated macrophages. Clin. Dev. Immunol..

[62] Lenos K., Goos J.A., Vuist I.M., den Uil S.H., Delis-van Diemen P.M., Belt E.J., Stockmann H.B., Bril H., de Wit M., Carvalho B. (2015). MGL ligand expression is correlated to BRAF mutation and associated with poor survival of stage III colon cancer patients. Oncotarget.

[63] Saeland E., van Vliet S.J., Backstrom M., van den Berg V.C., Geijtenbeek T.B., Meijer G.A., van Kooyk Y. (2007). The C-type lectin MGL expressed by dendritic cells detects glycan changes on MUC1 in colon carcinoma. Cancer Immunol. Immunother..

[64] Napoletano C., Rughetti A., Agervig Tarp M.P., Coleman J., Bennett E.P., Picco G., Sale P., Denda-Nagai K., Irimura T., Mandel U. (2007). Tumor-associated Tn-MUC1 glycoform is internalized through the macrophage galactose-type C-type lectin and delivered to the HLA class I and II compartments in dendritic cells. Cancer Res..

[65] Saeland E., Belo A.I., Mongera S., van Die I., Meijer G.A., van Kooyk Y. (2012). Differential glycosylation of MUC1 and CEACAM5 between normal mucosa and tumour tissue of colon cancer patients. Int. J. Cancer.

[66] Varki A. (2011). Since there are PAMPs and DAMPs, there must be SAMPs? Glycan "self-associated molecular patterns" dampen innate immunity, but pathogens can mimic them. Glycobiology.

[67] Crocker P.R., Paulson J.C., Varki A. (2007). Siglecs and their roles in the immune system. Nat. Rev. Immunol..

[68] Backer R., Schwandt T., Greuter M., Oosting M., Jungerkes F., Tuting T., Boon L., O'Toole T., Kraal G., Limmer A. (2010). Effective collaboration between marginal metallophilic macrophages and CD8^+^ dendritic cells in the generation of cytotoxic T cells. Proc. Natl. Acad. Sci. USA.

[69] Veninga H., Borg E.G., Vreeman K., Taylor P.R., Kalay H., van Kooyk Y., Kraal G., Martinez-Pomares L., den Haan J.M. (2015). Antigen targeting reveals splenic CD169^+^ macrophages as promoters of germinal center B-cell responses. Eur. J. Immunol..

[70] Nath D., Hartnell A., Happerfield L., Miles D.W., Burchell J., Taylor-Papadimitriou J., Crocker P.R. (1999). Macrophage-tumour cell interactions: identification of MUC1 on breast cancer cells as a potential counter-receptor for the macrophage-restricted receptor, sialoadhesin. Immunology.

[71] Toda M., Akita K., Inoue M., Taketani S., Nakada H. (2008). Down-modulation of B cell signal transduction by ligation of mucins to CD22. Biochem. Biophys. Res. Commun..

[72] Ishida A., Ohta M., Toda M., Murata T., Usui T., Akita K., Inoue M., Nakada H. (2008). Mucin-induced apoptosis of monocyte-derived dendritic cells during maturation. Proteomics.

[73] Ohta M., Ishida A., Toda M., Akita K., Inoue M., Yamashita K., Watanabe M., Murata T., Usui T., Nakada H. (2010). Immunomodulation of monocyte-derived dendritic cells through ligation of tumor-produced mucins to Siglec-9. Biochem. Biophys. Res. Commun..

[74] Hollingsworth M.A., Swanson B.J. (2004). Mucins in cancer: protection and control of the cell surface. Nat. Rev. Cancer.

[75] Lloyd K.O., Burchell J., Kudryashov V., Yin B.W., Taylor-Papadimitriou J. (1996). Comparison of O-linked carbohydrate chains in MUC-1 mucin from normal breast epithelial cell lines and breast carcinoma cell lines. Demonstration of simpler and fewer glycan chains in tumor cells. J. Biol. Chem..

[76] Michaelsson E., Broddefalk J., Engstrom A., Kihlberg J., Holmdahl R. (1996). Antigen processing and presentation of a naturally glycosylated protein elicits major histocompatibility complex class II-restricted, carbohydrate-specific T cells. Eur. J. Immunol..

[77] Haurum J.S., Arsequell G., Lellouch A.C., Wong S.Y., Dwek R.A., McMichael A.J., Elliott T. (1994). Recognition of carbohydrate by major histocompatibility complex class I-restricted, glycopeptide-specific cytotoxic T lymphocytes. J. Exp. Med..

[78] Vlad A.M., Muller S., Cudic M., Paulsen H., Otvos L., Hanisch F.G., Finn O.J. (2002). Complex carbohydrates are not removed during processing of glycoproteins by dendritic cells: processing of tumor antigen MUC1 glycopeptides for presentation to major histocompatibility complex class II-restricted T cells. J. Exp. Med..

[79] Ninkovic T., Hanisch F.G. (2007). O-glycosylated human MUC1 repeats are processed *in vitro* by immunoproteasomes. J. Immunol..

[80] Hanisch F.G., Schwientek T., von Bergwelt-Baildon M.S., Schultze J.L., Finn O. (2003). O-Linked glycans control glycoprotein processing by antigen-presenting cells: A biochemical approach to the molecular aspects of MUC1 processing by dendritic cells. Eur. J. Immunol..

[81] Hiltbold E.M., Vlad A.M., Ciborowski P., Watkins S.C., Finn O.J. (2000). The mechanism of unresponsiveness to circulating tumor antigen MUC1 is a block in intracellular sorting and processing by dendritic cells. J. Immunol..

[82] Madsen C.B., Petersen C., Lavrsen K., Harndahl M., Buus S., Clausen H., Pedersen A.E., Wandall H.H. (2012). Cancer associated aberrant protein O-glycosylation can modify antigen processing and immune response. PLoS ONE.

[83] Singh S.K., Streng-Ouwehand I., Litjens M., Kalay H., Saeland E., van Kooyk Y. (2011). Tumour-associated glycan modifications of antigen enhance MGL2 dependent uptake and MHC class I restricted CD8 T cell responses. Int. J. Cancer.

[84] Monti P., Leone B.E., Zerbi A., Balzano G., Cainarca S., Sordi V., Pontillo M., Mercalli A., di Carlo V., Allavena P. (2004). Tumor-derived MUC1 mucins interact with differentiating monocytes and induce IL-10^high^IL-12^low^ regulatory dendritic cell. J. Immunol..

[85] Rughetti A., Pellicciotta I., Biffoni M., Backstrom M., Link T., Bennet E.P., Clausen H., Noll T., Hansson G.C., Burchell J.M. (2005). Recombinant tumor-associated MUC1 glycoprotein impairs the differentiation and function of dendritic cells. J. Immunol..

[86] Von Mensdorff-Pouilly S., Petrakou E., Kenemans P., van Uffelen K., Verstraeten A.A., Snijdewint F.G., van Kamp G.J., Schol D.J., Reis C.A., Price M.R. (2000). Reactivity of natural and induced human antibodies to MUC1 mucin with MUC1 peptides and n-acetylgalactosamine (GalNAc) peptides. Int. J. Cancer..

[87] Blixt O., Bueti D., Burford B., Allen D., Julien S., Hollingsworth M., Gammerman A., Fentiman I., Taylor-Papadimitriou J., Burchell J.M. (2011). Autoantibodies to aberrantly glycosylated MUC1 in early stage breast cancer are associated with a better prognosis. Breast Cancer Res..

[88] Kumamoto Y., Linehan M., Weinstein J.S., Laidlaw B.J., Craft J.E., Iwasaki A. (2013). CD301b^+^ dermal dendritic cells drive T helper 2 cell-mediated immunity. Immunity.

[89] Freire T., Zhang X., Deriaud E., Ganneau C., Vichier-Guerre S., Azria E., Launay O., Lo-Man R., Bay S., Leclerc C. (2010). Glycosidic Tn-based vaccines targeting dermal dendritic cells favor germinal center B-cell development and potent antibody response in the absence of adjuvant. Blood.

[90] Chen H.D., Zhou X., Yu G., Zhao Y.L., Ren Y., Zhou Y.D., Li Q., Zhang X.L. (2012). Knockdown of core 1 beta 1, 3-galactosyltransferase prolongs skin allograft survival with induction of galectin-1 secretion and suppression of CD8^+^ T cells: T synthase knockdown effects on galectin-1 and CD8^+^ T cells. J. Clin. Immunol..

[91] Gringhuis S.I., den Dunnen J., Litjens M., van der Vlist M., Geijtenbeek T.B. (2009). Carbohydrate-specific signaling through the DC-SIGN signalosome tailors immunity to Mycobacterium tuberculosis, HIV-1 and Helicobacter pylori. Nat. Immunol..

[92] Van Vliet S.J., Vuist I.M., Lenos K., Tefsen B., Kalay H., Garcia-Vallejo J.J., van Kooyk Y. (2013). Human T cell activation results in extracellular signal-regulated kinase (ERK)-calcineurin-dependent exposure of Tn antigen on the cell surface and binding of the macrophage galactose-type lectin (MGL). J. Biol. Chem..

[93] Li D., Romain G., Flamar A.L., Duluc D., Dullaers M., Li X.H., Zurawski S., Bosquet N., Palucka A.K., Le Grand R. (2012). Targeting self- and foreign antigens to dendritic cells via DC-ASGPR generates IL-10-producing suppressive CD4^+^ T cells. J. Exp. Med..

[94] Napoletano C., Zizzari I.G., Rughetti A., Rahimi H., Irimura T., Clausen H., Wandall H.H., Belleudi F., Bellati F., Pierelli L. (2012). Targeting of macrophage galactose-type C-type lectin (MGL) induces DC signaling and activation. Eur. J. Immunol..

[95] Carrascal M.A., Severino P.F., Guadalupe Cabral M., Silva M., Ferreira J.A., Calais F., Quinto H., Pen C., Ligeiro D., Santos L.L. (2014). Sialyl Tn-expressing bladder cancer cells induce a tolerogenic phenotype in innate and adaptive immune cells. Mol. Oncol..

[96] Perdicchio M., Cornelissen L.A., Streng-Ouwehand I., Engels S., Verstege M.I., Boon L., Geerts D., van Kooyk Y., Unger W.W. (2016). Tumor sialylation impedes T cell mediated anti-tumor responses while promoting tumor associated-regulatory T cells. Oncotarget.

[97] Perdicchio M., Ilarregui J.M., Verstege M.I., Cornelissen L.A., Schetters S.T., Engels S., Ambrosini M., Kalay H., Veninga H., den Haan J.M. (2016). Sialic acid-modified antigens impose tolerance via inhibition of T-cell proliferation and de novo induction of regulatory T cells. Proc. Natl. Acad. Sci. USA.

[98] Ando M., Tu W., Nishijima K., Iijima S. (2008). Siglec-9 enhances IL-10 production in macrophages via tyrosine-based motifs. Biochem. Biophys. Res. Commun..

[99] Takamiya R., Ohtsubo K., Takamatsu S., Taniguchi N., Angata T. (2013). The interaction between Siglec-15 and tumor-associated sialyl-Tn antigen enhances TGF-beta secretion from monocytes/macrophages through the DAP12-Syk pathway. Glycobiology.

[100] Morizane T., Watanabe T., Tsuchimoto K., Tsuchiya M. (1980). Impaired T cell function and decreased natural killer activity in patients with liver cirrhosis and their significance in the development of hepatocellular carcinoma. Gastroenterol. Jpn..

[101] Sullivan J.L., Byron K.S., Brewster F.E., Purtilo D.T. (1980). Deficient natural killer cell activity in x-linked lymphoproliferative syndrome. Science.

[102] Van Rinsum J., Smets L.A., van Rooy H., van den Eijnden D.H. (1986). Specific inhibition of human natural killer cell-mediated cytotoxicity by sialic acid and sialo-oligosaccharides. Int. J. Cancer.

[103] Ogata S., Maimonis P.J., Itzkowitz S.H. (1992). Mucins bearing the cancer-associated sialosyl-Tn antigen mediate inhibition of natural killer cell cytotoxicity. Cancer Res..

[104] Cohen M., Elkabets M., Perlmutter M., Porgador A., Voronov E., Apte R.N., Lichtenstein R.G. (2010). Sialylation of 3-methylcholanthrene-induced fibrosarcoma determines antitumor immune responses during immunoediting. J. Immunol..

[105] Jandus C., Boligan K.F., Chijioke O., Liu H., Dahlhaus M., Demoulins T., Schneider C., Wehrli M., Hunger R.E., Baerlocher G.M. (2014). Interactions between Siglec-7/9 receptors and ligands influence NK cell-dependent tumor immunosurveillance. J. Clin. Invest..

[106] Hudak J.E., Canham S.M., Bertozzi C.R. (2014). Glycocalyx engineering reveals a Siglec-based mechanism for NK cell immunoevasion. Nat. Chem. Biol..

